# Microscopic Structure of Swollen Hydrogels by Scanning Electron and Light Microscopies: Artifacts and Reality

**DOI:** 10.3390/polym12030578

**Published:** 2020-03-05

**Authors:** Zhansaya Kaberova, Evgeny Karpushkin, Martina Nevoralová, Miroslav Vetrík, Miroslav Šlouf, Miroslava Dušková-Smrčková

**Affiliations:** 1 Institute of Macromolecular Chemistry, Academy of Sciences of the Czech Republic, Heyrovského náměstí 2, 162 06 Praha 6, Prague, Czech Republic; kaberova@imc.cas.cz (Z.K.); nevoralova@imc.cas.cz (M.N.); vetrik@imc.cas.cz (M.V.); slouf@imc.cas.cz (M.Š.); 2 Department of Chemistry, Lomonosov Moscow State University, 119991 Moscow, Russia; eukarr@gmail.com

**Keywords:** hydrogel, PHEMA, poly(2-hydroxyethyl methacrylate), poly(glycerol monomethacrylate), PGMA, morphology, variable-pressure and environmental scanning electron microscopy, laser scanning confocal microscopy, artifacts

## Abstract

The exact knowledge of hydrogel microstructure, mainly its pore topology, is a key issue in hydrogel engineering. For visualization of the swollen hydrogels, the cryogenic or high vacuum scanning electron microscopies (cryo-SEM or HVSEM) are frequently used while the possibility of artifact-biased images is frequently underestimated. The major cause of artifacts is the formation of ice crystals upon freezing of the hydrated gel. Some porous hydrogels can be visualized with SEM without the danger of artifacts because the growing crystals are accommodated within already existing primary pores of the gel. In some non-porous hydrogels the secondary pores will also not be formed due to rigid network structure of gels that counteracts the crystal nucleation and growth. We have tested the limits of true reproduction of the hydrogel morphology imposed by the swelling degree and mechanical strength of gels by investigating a series of methacrylate hydrogels made by crosslinking polymerization of glycerol monomethacrylate and 2-hydroxyethyl methacrylate including their interpenetrating networks. The hydrogel morphology was studied using cryo-SEM, HVSEM, environmental scanning electron microscopy (ESEM), laser scanning confocal microscopy (LSCM) and classical wide-field light microscopy (LM). The cryo-SEM and HVSEM yielded artifact-free micrographs for limited range of non-porous hydrogels and for macroporous gels. A true non-porous structure was observed free of artifacts only for hydrogels exhibiting relatively low swelling and high elastic modulus above 0.5 MPa, whereas for highly swollen and/or mechanically weak hydrogels the cryo-SEM/HVSEM experiments resulted in secondary porosity. In this contribution we present several cases of severe artifact formation in PHEMA and PGMA hydrogels during their visualization by cryo-SEM and HVSEM. We also put forward empirical correlation between hydrogel morphological and mechanical parameters and the occurrence and intensity of artifacts.

## 1. Introduction

In tissue engineering, both non-porous as well as porous hydrophilic methacrylate hydrogels are widely used [1,2,3,4,5]. For eye lens implants or contact lenses, hydrogels must be optically homogeneous and thus they must not contain any light-scattering objects such as pore walls or supramolecular inhomogeneity formed by polymer chain associations [6]. Nanodomains of size up to several tens of nanometers formed for example by polymer chain organization or resulting from local density fluctuations [7,8] might be acceptable. Another class of hydrogels comprises heterogeneous porous materials designed for biological applications, e.g., for cell culture where the internal microstructure such as the size, shape and communication of pores are the major parameters determining the biological response. Therefore, morphological studies of hydrogels are very important and frequent and gel images obtained by number of microscopy methods—perhaps SEM-based methods most frequently—are typically included in publications on gels.

It is indeed essential to obtain true information about the morphology of hydrogels in their native swollen state that is relevant to gel application conditions. Several powerful techniques able to handle swollen gels are available to serve: light microscopy (LM), laser scanning confocal microscopy (LSCM) and micro computed tomography (micro-CT) [9,10] but these techniques have limited resolution (LM, micro-CT), limited depth of field (LSCM) and/or they demand labeling or contrasting the hydrogel chemically (LSCM, micro-CT). Indeed, a major class of methods are the scanning electron microscopies (SEM), particularly high vacuum SEM (HVSEM), cryogenic low vacuum SEM (cryo-LVSEM or cryo-SEM) and environmental scanning SEM (ESEM), which provide information about structure that can be obtained on sub-micron level and with a large depth of field. However, a critical step in hydrogel characterization by SEM is the inevitable solidification using drying or freezing of the water-swollen material to suppress water evaporation in the microscope chamber. Typically, plunge-freezing of a swollen sample done either under normal pressure or under high pressure is employed [11]. The involved temperature changes cause a transition of the swollen gel system to a solid state while increasing the likelihood of change of sample morphology. For instance, the difference in morphology of macroporous poly(2-hydroxyethyl methacrylate) (PHEMA) hydrogels was observed when characterized in the dry state by SEM and in the ambient swollen state by LSCM [12]. Different ways of dehydration caused changes in morphology to different extents. When freeze-drying after the plunge-freezing in liquid nitrogen at −196 °C was used, the native morphology of PHEMA samples was preserved more accurately as compared to the freeze-drying after freezing at −20 °C followed by critical point drying using the acetone/CO_2_ (liquid) system [12]. For poly(vinyl alcohol) (PVA) hydrogels, the plunge-freezing in liquid nitrogen and critical point drying in the ethanol/CO_2_ (liquid) system revealed different surface and bulk morphology by SEM [13]. The morphology of copolymers of vinyl alcohol and *N*-vinyl-2-pyrrolidone hydrogels freeze-dried at 5 μm Hg and −23 °C before SEM analysis differed significantly from morphology of the samples placed into SEM chamber in the frozen state. The ice crystals sublimed in the microscope during observation formed time-dependent heterogeneous patterns [14]. This so called “freeze-etching” caused by heating from the electron beam promoted sublimation of the diluent under low pressure and resulted in structure failure of swollen samples. Alginate hydrogels with different water content were comprehensively characterized by cryo-SEM [15] involving plunge-freezing using liquid nitrogen slush (*T* = −210 °C), liquid ethane and high–pressure freezing techniques. It was shown that the plunge-freezing provided true alginate hydrogels structure only in a region close to the sample edges (20 μm) [15]. The high-pressure freezing at 2100 bar involved notably higher cooling rate and heat-transfer coefficient and it prevented the formation of hexagonal ice crystal within the gel bulk [15].

The application of other, well-established microscopic techniques to hydrogels is rather limited due to specific properties of these materials [16]: transmission electron microscopy (TEM) is not suitable because the preparation ultrathin sections (thickness 50 nm) without artifacts is almost impossible, atomic force microscopy (AFM) is impractical due to hydrogel roughness (limited Z-range of AFM) and porosity (limited XY-range of AFM) and applications of X-ray microtomography (micro-CT) on low-contrast polymer materials are still under development.

The strategies used to produce defined porosity of gel scaffolds include the use of sacrificed templates [17,18], phase separation during gelation [19,20], in situ crystallization of diluent upon freezing (cryogelation) [21] or more recently 3D printing approaches such as direct writing of photopolymerizable hydrophilic polymers [22].

Both hydrophilic polymethacrylates poly(2-hydroxyethyl methacrylate), PHEMA, and poly(glycerol monomethacrylate), PGMA, are biocompatible materials and besides their major long term application in ophthalmology they are also often used as media for cell culture and growth [2,23]. For proper application in these fields, morphology of hydrogels must be precisely controlled. It can be done through varying the ratio of components in the reaction mixture [24]: for example, certain excess of diluent can trigger pore formation during polymerization by reaction induced phase separation [19]. For example, lightly crosslinked systems based on PHEMA undergo the phase separation above the critical amount of water about 40–45 vol.% [3,20,25]. PGMA hydrogels are more hydrophilic and thus they undergo phase separation leading to macroporous morphology only at high water content: higher than 90 vol.%. This fact follows from the plot of water content in monomer mixture vs. water content in swollen hydrogel shown in [26]. Woerly at al. observed porosity in PGMA gels when prepared at the water level in the monomer mixture 60–80 vol.% and with low crosslinker content [27]. Indeed, in the PGMA mixtures with less hydrophilic monomers, porosity caused by phase separation appeared at a lower water level such as 50 vol.%, as found for the example in [28]. In these papers, morphology of hydrogels was studied by scanning electron microscopy methods that involved sample plunge-freezing in liquid nitrogen and subsequent freeze-drying.

Despite the large number of papers dealing with the structural artifacts observed in hydrogels subjected to various types of electron microscopy techniques (involving the exposition of gels to various freezing conditions applied during the sample preparation steps) at least in part discussed above, the issue of correct, artifact-free visualization of swollen hydrogels still remains open. It is relatively well-known that hydrogels containing a large amount of water upon freezing are prone to show artifacts caused by mechanical deformation or even failure of the soft gel by developing ice crystals. Hydrogels with a heterogeneous porous topology show different behavior: if the size and number of pores are sufficient, the ice crystals start growing from “free water” in the pores where they are accommodated often without deforming the surrounding gel. It has so far not been clarified to which swelling extent and for which mechanical properties and chemical compositions of gels and in combination with which preparation techniques the artifacts can be expected.

**Scheme 1:**
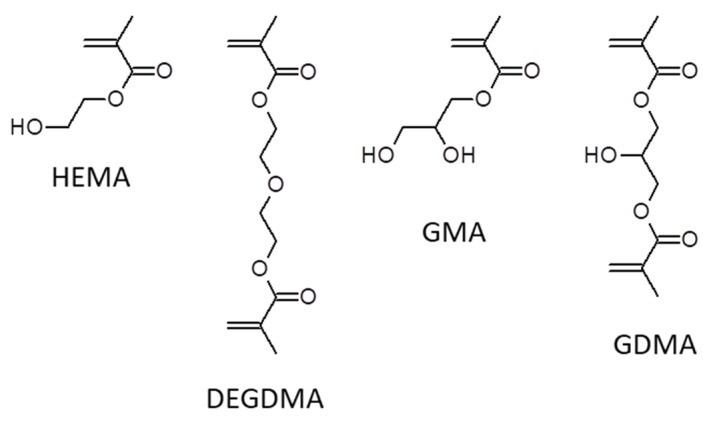
Structural formulas of the monomers and crosslinkers used for synthesis of hydrogels. HEMA, 2-hydroxyethyl methacrylate; DEGDMA, di(ethylene glycol) dimethacrylate; GMA, glycerol monomethacrylate and GDMA, glycerol dimethacrylate.

In our work, we have addressed the question of the limits for correct hydrogel visualization by SEM-based imaging methods in terms of hydrogel swelling degree and mechanical strength to which these methods still work reliably. To answer the question, we have prepared a series of methacrylate-based hydrogels with different equilibrium swelling in water and different mechanical strengths: from soft, highly swollen to stiff, low-swollen species. Within this series, a morphology of hydrogels differed from homogenous, non–macroporous to microstructured by reaction-induced phase separation during crosslinking polymerization. The monomers of choice were glycerol methacrylate (GMA) and 2-hydroxyethyl methacrylate (HEMA), crosslinked with their chemically close analogues, di(ethylene glycol) dimethacrylate (DEGDMA); and glycerol dimethacrylate (GDMA). The chemical structures of monomers are shown in Scheme 1. The gels were either single networks or interpenetrating networks (IPNs hydrogels) with reinforced structures of both homogeneous and heterogeneous morphologies. The formed hydrogels were studied by both electron and light microscopies. We have employed the method of cryogenic low-vacuum scanning electron microscopy (cryo-SEM) where the samples were plunge-frozen in liquid nitrogen (LN_2_). As a next immediate the still frozen samples were scanned directly omitting the lyophilizing step. Beside this technique, environmental scanning electron microscopy (ESEM) with no exposition of the samples to sub-zero temperatures was used.

To visualize the real structures of swollen hydrogels and identify a possible secondary porosity caused by SEM-required treatments, laser scanning confocal microscopy (LSCM) and classical wide-field light microscopy (LM) were used. In this work, we show the decisive impact of the process of sample freezing, and suggest the selection of suitable artifact-free observation methods for swollen hydrogels. In addition, we were able to link the swelling extent of gels and mechanical stiffness (described here using low strain elastic moduli) of studied hydrogels with semi-quantitative relation to the appearance of artifacts using the cryo-SEM technique.

## 2. Experimental

### 2.1. Chemicals

Monomers glycerol monomethacrylate, GMA, (BASF, Ludwigshafen, Germany), 2-hydroxyethyl methacrylate, HEMA, (Rohm and Haas, Philadelphia, USA), di(ethylene glycol) dimethacrylate, DEGDMA, (Sigma-Aldrich, Saint Louis, MO, USA – now Merck, Darmstadt, Germany) and glycerol dimethacrylate, GDMA, (Sigma-Aldrich) were used as received. The redox initiator system was composed of ammonium persulfate APS (Fluka Chemie, Buchs, Switzerland), *N*,*N*,*N’*,*N’*-tetramethylethylenediamine TEMED (Sigma-Aldrich) and UV-initiator 2-hydroxy-2-methyl-1-phenyl-propane-1-one, brand name Ciba® DAROCUR® 1173 (Sigma-Aldrich). Fluorescein isothiocyanate isomer I (FITC), DY-677-NHS (amine coupling asymmetric cyanine ester) and *N*-(3-aminopropyl) methacrylamide hydrochloride (methacryloylating agent) were purchased from Sigma-Aldrich and further chemically modified to introduce polymerizable groups utilized in gel structure visualization by laser scanning confocal microscopy.

### 2.2. Synthesis of Hydrogels

The hydrogels were synthesized using free-radical polymerization initiated by the redox system at room temperature either in the presence or absence of water as a diluent. Details of synthesis are given in Supplementary information. The list of produced sample, and their description including swelling and mechanical parameters is presented in Table 1.

### 2.3. Coding of Samples

The single network gels were coded as, for example, H80/1 or G0/0.3. Here, the first letter (H and G) means the HEMA or GMA monomer, the numbers following the letter define the concentration of water used as a diluent at polymerization in vol.% and the numbers following the slash express the amount of added crosslinker DEGDMA in mol% per monomers (monomers composed of monomer and crosslinker). Note, that there was always a small additional constant amount of EGDMA (0.2 wt%) or glycerol dimethacrylate (0.2 wt%) that will act as a crosslinker present in the HEMA and GMA monomers respectively. The IPN hydrogels were coded as, for example, H80/1–G0/0.3 where the first part describes the composition of the first network, and the part following the hyphen describes the composition of the second network.

### 2.4. Electron Microscopy

Morphology of hydrogels was studied by three different electron microscopy techniques: classical, high-vacuum scanning electron microscopy (HVSEM or briefly SEM), cryogenic low-vacuum scanning electron microscopy (cryo-LVSEM or briefly cryo-SEM) and environmental scanning electron microscopy (ESEM). It is worth mentioning that LVSEM and ESEM techniques are sometimes referred to as variable-pressure microscopy (VP-SEM). All three techniques were carried out using a Quanta 200 FEG microscope (FEI, Czech Republic), which was equipped by detectors for all three modes (high-vacuum, low-vacuum and environmental mode) and a Peltier cooling stage (allowing to decrease specimen temperature down to −20 °C).

Standard HVSEM microscopy and lyophilization of swollen gels. HVSEM can be applied only to dry, conductive specimens in vacuum. Therefore, before the observation in HVSEM, the hydrogel samples had to be dried by lyophilization (sample 2 mm × 2 mm × 2 mm first frozen in a refrigerator at −30 °C for three hours and then freeze-dried for 12 h at −90 °C at 0.1 mbar using lyophilizer Gregor Instrument). Then, the sample was attached by conductive adhesive tape (double adhesive carbon tape; Christine Groepl, Austria) to an aluminum specimen holder, and covered with a thin platinum layer (vacuum sputter coater SCD 050; Leica, Austria). Then the samples could be inserted in the SEM microscope and observed in high vacuum using a secondary electron detector at accelerating voltage 10 kV.

Cryo-LVSEM microscopy can visualize specimens containing frozen water. In a typical Cryo-LVSEM experiment, a small piece of the hydrogel sample (ca 2 mm × 2 mm × 2 mm) was quickly frozen in liquid nitrogen and transferred to a Peltier Specimen Stage (cooled to −10 °C) in a vacuum chamber of the microscope. The frozen specimen was fixed on a Peltier specimen stage with a droplet of water (the water drop freezes within a few seconds while fixing the sample to the stage) and a thin upper layer of the specimen was cut off with a liquid-nitrogen cooled razor blade (this removes the upper layer of frozen specimen, which can be deformed and/or covered with ice microcrystals). The morphology of the hydrogel was observed in a low-vacuum mode (chamber pressure 80–100 Pa) using a low-vacuum secondary electrons detector at an accelerating voltage of 10 kV.

ESEM microscopy can visualize the morphology of swollen specimens, close to their native state in solution, and avoids the freezing step (which might cause the ice-crystallization leading to gel structure changes). A small piece of hydrogel (ca 2 mm × 2 mm × 2 mm) was cut with a sharp blade at ambient temperature and placed in a water containing sample holder, which was incorporated at the Peltier stage whose temperature was kept at +1 °C during the whole experiment. The chamber pressure was set to 700 Pa and the specimen was inserted. Under these conditions it was possible to achieve 100% humidity in the chamber of the ESEM microscope (700 Pa is higher than equilibrium vapor pressure of water at +1 °C). A water film was removed from the surface layer of the specimen by evaporation through controlled decrease in the chamber pressure (typical chamber pressures during the experiment range from 300 to 700 Pa). The specimen was observed at accelerating voltage of 30 kV using ESEM detector; the micrographs of the swollen hydrogel were recorded at temperature/pressure +1 °C/300–700 Pa and humidity close to 100%, as explained above.

### 2.5. Wide-Field Light Microscopy

Classical wide-field light microscopy (LM) observations were performed using a Leica DM6000 M microscope (Leica, Austria) equipped with extra-large working distance objectives with a high depth of focus. For the LM experiment, a thin wedge-shaped section of the hydrogel was prepared by cutting a larger specimen with a sharp razor blade, a wedge was placed on the microscopic support glass with a few drops of water and covered with a thin microscopic cover glass. During the whole sample preparation and microscopic observations, the sample was kept fully submerged in water at ambient conditions, i.e., the gel was at the equilibrium swollen state. The microscopy images were taken at room temperature in either transmitted light (diascopic illumination) or reflected light (episcopic illumination) mode by means of both bright- and dark-field imaging.

### 2.6. Laser Scanning Confocal Microscopy

The structure of equilibrium swollen hydrogels at ambient conditions was visualized by laser scanning confocal microscopy (LSCM) using a Fluoview FV1200 instrument (Olympus, Japan). An argon ion laser and a diode laser providing emissions at wavelengths of 488 and 635 nm were used to visualize the networks covalently labeled with methacryloylated FITC and DY 677, respectively. Fluorescence images were captured with a 50× objective and double zoom, at room temperature. Hydrogels for visualization by LSCM were covalently modified with fluorescent dyes. The procedures of chemical modification of the dyes as well as of the preparation of dye-modified hydrogels are described in Supporting information.

### 2.7. Equilibrium Water Content

Swelling of hydrogels was determined gravimetrically. A disc-shaped specimen of a swollen hydrogel was weighed and dried in a vacuum oven at 100 °C to constant mass. Equilibrium water content, EWC (g/g), of the hydrogels was determined as:

(1) $$\mathrm{EWC}=\frac{m_{\mathrm{sw}}-m_{d}}{m_{d}}$$

where *m*_sw_ and *m*_d_ are masses of the swollen and dry hydrogel, respectively. If the solvent other than water was used to determine swelling, the liquid content was calculated analogically and called ESC (equilibrium solvent content).

### 2.8. Tensile Measurements

Tensile mechanical measurements were performed using a micro-tensile instrument Linkam TST 350 with maximum load of 50 N with equilibrium swollen samples at ambient temperature. All hydrogels were tested after complete equilibration immersed in distilled water. The samples were patterned into dog-bone shaped pieces of 8 mm × 4 mm (central part). The specimen thickness varied between 1 and 3 mm, depending on its swelling capacity. Hydrogels were fixed in the grips, and the extension speed was 10 μm/s. For each hydrogel composition, at least five specimens were measured, and the results were averaged.

## 3. Results and Discussion

### 3.1. Microstructure of Highly Swollen Homogeneous PGMA Hydrogel 

**Figure 1:**
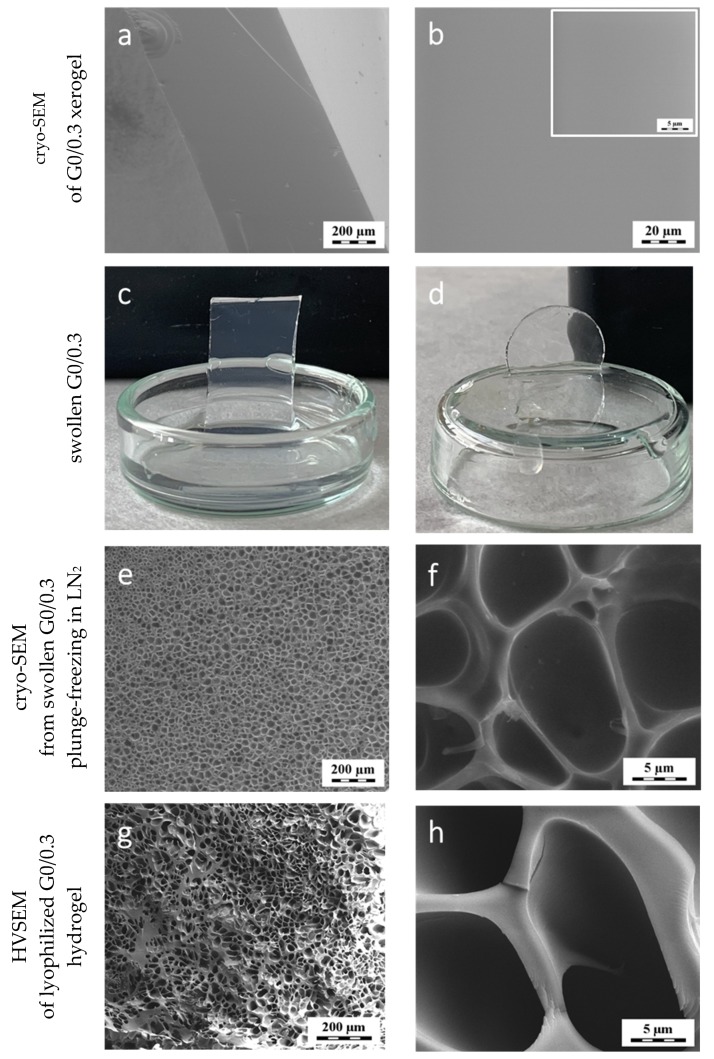
Appearance of the G0/0.3 sample at different stages: (**a**,**b**) G0/0.3 xerogel observed in the dry state as obtained by bulk polymerization; (**c**,**d**) photographs of the equilibrium water- and DMSO-swollen G0/0.3 hydrogel, respectively, (**e**,**f**) cryo-SEM from the water-swollen sample and (**g**,**h**) high vacuum scanning electron microscopies (HVSEM) of the lyophilized sample.

The polyglycerol-based homogeneous, i.e., non-macroporous methacrylate hydrogel was prepared by crosslinking polymerization in bulk while macroscopically clear transparent resin not revealing any microscopic structure by cryo-SEM (Figure 1a,b) was formed. The gel transparency was maintained in its equilibrium swollen state in water at which the gel contained 2.8 g of water per gram of dry resin. Under closer inspection with the naked eye against a dark background, the water-swollen specimen revealed a very slight hint of scattering of light that disappeared when the swollen gel was even more expanded by swelling in DMSO to ESC = 5.6 g (Figure 1c,d, respectively).

Cryo-SEM was our first choice to check the water-swollen G0/0.3 sample structure; the sample preparation for cryo-SEM involved sample plunge-freezing in LN_2_ and no subsequent freeze-drying. Surprisingly, the cryo-SEM micrographs revealed severe heterogeneity in the form of cellular pores of 5–40 μm distributed over the entire cross-cut sample surface, see images in Figure 1e,f. Similar heterogeneity but with somewhat larger pores and wider distribution of pore sizes than was showed on cryo-SEM images, was revealed by the HVSEM method where samples had to be Pt-coated after being frozen in the refrigerator and freeze-dried, cf. images g and h in Figure 1. 

The above mentioned minor light scattering from swollen sample makes one think of a possible optical heterogeneity: some initially “white” gels upon swelling may become clear because the refractive indices of light in the gel and in the swelling medium become very similar. The PGMA xerogel resin after polymerization readily swelled in the GMA monomer—the ESC for GMA in polyGMA was 1.7 g/g (RT experiment)—in other words, more of the monomer was absorbed by its polymer homologue. This swelling capacity suggested that the phase separation during polymerization was not in play in this case: otherwise, the polymer would not be compatible with more of its monomer and would not swell in it. Thus, the produced resin is homogeneous similarly to resins of poly(methylmethacrylate). The SEM investigation also showed that structural inhomogeneities on the order of tens of micrometers did not exist in the PGMA xerogel right after polymerization (cf. Figure 1a–d).

The next source of heterogeneities detected by cryo-SEM and HVSEM could stem from the preparation of sample for cryo-SEM by fast-freeze process. It is commonly assumed that the plunge-freezing of hydrated samples should preserve their structure [27,28,29] or could induce formation of only submicron size pores easily identified as artifacts due to incipient ice formation. Such processes are in detail discussed by Paterson et al. [12] in the case of poly[HEMA-*co*-MeO-PEGMA] hydrogels, that are chemically similar to PGMA and swell more than the neat PHEMA gel, these gels contained even more water during their plunge-freezing in LN_2_—around 85 wt% than our PGMA sample (74 wt.% of water) showed no significant change of structure, cf. Figure 3g in reference [12].

Other reasons of the formation of such a heterogeneous structure like the one seen on Figure 1e,f considered by us were (a) formation of bubbles during polymerization possibly from dissolved air or nitrogen used for the purging of reactants, and (b) heterogeneity caused by a possible only partial compatibility of the di(ethylene glycol) dimethacrylate crosslinker with the glycerol monomethacrylate monomer (cf. structures in Scheme 1). To confirm these hypotheses, we synthesized the PGMA gels again under several sets of conditions (described in detail in the Supplementary information). These conditions included (a) thorough degassing of the polymerization mixture while still DEGDMA was used as the crosslinker, (b) all monomers were freshly distilled prior to polymerization, then degassed and again DEGDMA was used as a crosslinker and (c) monomers were degassed before and after distillation, and the crosslinker was glycerol dimethacrylate, GDMA, as the closest chemical analogue to GMA (cf. Scheme 1) to ensure good miscibility. Although, such heterogeneous structure as shown in Figure 1e,f should be detectable in the as prepared polymer system even before its swelling, the resulting resins were always glassy, fully transparent and in cryo-SEM images did not show any signs of such structural elements, cf. Figure 1a,b.

**Figure 2:**
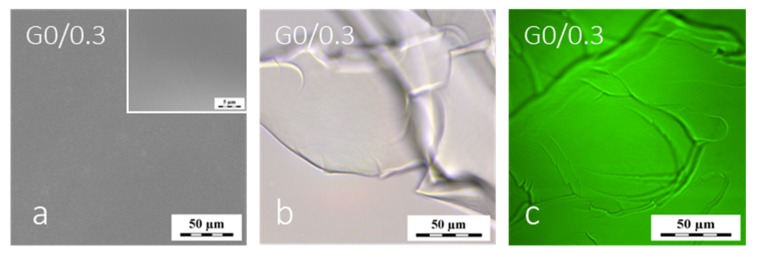
Morphology of the G0/0.3 hydrogel swollen in water observed by methods: (**a**) environmental scanning SEM (ESEM), (**b**) light microscopy (LM) and (**c**) laser scanning confocal microscopy (LSCM).

To track the effect of the plunge-freezing step and treatment under low temperatures at cryo-SEM on the structure of the sample G0/0.3, we also employed methods where no gel freezing was involved: such as the environmental scanning electron microscopy (ESEM). ESEM that does include sample cooling but only to temperatures above the freezing point of water. Indeed, the depression of the freezing point of water in the three-dimensional macromolecular network is involved, too, but discussion of this classical phenomena goes beyond the scope of this work, the reader may start from here [30]. The gels during ESEM characterization were cooled down to 2 °C. The light microscopy (LM) and finally the laser scanning confocal microscopy (LSCM) using a fluorescently labeled hydrogel were performed with swollen GMA samples at ambient conditions. The results are shown in Figure 2.

It should be noted that in ESEM, optimization of the chamber pressure is a very important parameter—if the appropriate pressure is not met, the sample surface structure may be flooded by a layer of liquid water (chamber temperature is +2 °C) or the surface structure may collapse by excessive drying (cf. Supplementary information, Figure S2). Therefore, in this ESEM experiment, the G0/0.3 gel was investigated under a range of pressures 360–660 Pa. Neither the ESEM nor the light microscopy images revealed a heterogeneous structure of this hydrogel (Figure 2a,b). However, the values of refractive indices of water and the water-swollen PGMA hydrogel were very close (1.333 and 1.366 respectively); hence, possible pores would be hardly observed with LM due to the lack of an optical contrast between the water-filled pores and water-swollen matrix. To resolve the issue of swollen structure existence, the PGMA hydrogel was covalently labeled with a modified fluorescein dye (details of the labeling procedure in the Supplementary information) and observed using the ambient LSCM. Since after the polymerization, the possible unpolymerized species (including the dye residues) were washed out of the hydrogel, the dye could be present only in the swollen gel phase, but not in water filling the pores (if any). The detected structure was evenly distributed over the entire sample space—see Figure 2c, where only the darker lines were caused by an imperfect surface of the specimen. In LSCM, the water-swollen G0/0.3 gel was found to be non-porous down to the distinguishable limit of units of micrometers. The evidence that the LSCM method has the capacity to clearly reveal a macroporous structure of fluorescently labeled swollen gels was also acquired and will be discussed in detail and illustrated later (gel H80/1 shown on Figure 5f).

**Figure 3:**
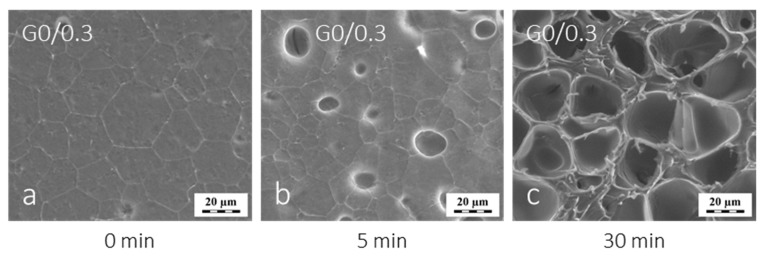
Effect of the time of exposition to electrons in the microscope chamber in cryo-SEM experiment on development of secondary pores in plunge-frozen water-swollen G0/0.3 hydrogel. (**a**) Initial specimen covered with a layer of frozen water (t ~ 0 min), (**b**) sublimation of ice in progress (t ≈ 5 min) and (**c**) complete sublimation of ice from the surface of the scanned area (t ≈ 30 min).

In summary, based on the ESEM, LM and LSCM observations, the swollen G0/0.3 hydrogel did not contain pores like those shown in Figure 1e,f. Clearly, these structures were secondary pores formed by crystallization of water in the gel and subsequent crystals sublimation under the chamber vacuum. Interestingly, the secondary cellular-like macropores in polyGMA gels were formed with great reproducibility always when water-swollen gels were treated by plunge-freezing and observed with cryo-SEM. We followed the development of secondary pores with observation time, see Figure 3. Initially, a patterned surface with just a few circular holes was seen (Figure 3a). The hexagonal pattern appeared within a frozen layer of water. After some time, more holes start forming in the hexagonal shape corners (Figure 3b). After some more time under the chamber conditions, more cavities appeared on the observed surface, and finally turned into cellular-like structures (Figure 3c). Similar effects of the prolonged exposure to the reduced pressure, and to the electron beam on the morphology of hydrogels have already been reported [14,15].

The structure of a hydrated sample treated by “cryo-fixation” strongly depends on the cooling rate at each volume element of the body that is related to its distance from the surface [31]. To preserve the swollen structure morphology, ideally, the cooling rate should be high enough to prevent crystallization of water and growth of crystals: water should attain its amorphous form. From the experimental studies with hydrogels, it follows that the plunge-freezing technique (plunging in LN_2_) under normal pressure can lead to structure preservation only when samples are very thin (10–40 μm) [11,32]. If a thin specimen of gel cannot be prepared, freezing should be performed under external pressure high enough to counteract the ice crystal growth (typically 2100 bar) [33,34]. The swelling degree of PGMA gels in water is quite high (EWC 2.8–5.8 g/g depending on gel dilution at polymerization) and the “standard procedure” of cooling by quick immersion of a small piece of the gel in LN_2_ when high pressure freeze is not involved results in the development of a new porous structure. This structure was linked to the formation of ice crystals [34,35]. Within observation time, these crystals may sublime and leave the empty pores in the material surface. Mechanism of this process in homogenous swollen methacrylate-based hydrogels was qualitatively described in the literature [28]. Quantitative features of the developing structure such as the average size and total volume of pores depend on the hydrogel swelling degree and dynamics of the freezing cycle.

During the observation in a cryo-SEM chamber, the frozen specimen was subjected to the electron beam irradiation under low vacuum (around 100 Pa), and the etching of the specimen occurred via the sublimation of ice crystals together with freeze drying of the hydrogel. The resulting voids could not be refilled with the network material, since the dehydrated gel under the observation conditions was a glassy material. When reswollen, the pores in the gel might partly recover.

### 3.2. PGMA Hydrogels Prepared in the Presence of Water as a Diluent

When gelation in the macromolecular system proceeds in a diluent, the resulting material is a swollen gel. If the swelling capacity of the gel macromolecular network is exceeded already during polymerization, the network will expel the additional liquid, i.e., it will phase separate and a heterogeneous material will be formed showing a typical microstructure: for example 2-hydroxyethyl methacrylate polymerized in water presence above 50 vol.% attains a well-known fused-sphere-like microstructure [3] with hydrogel spheres connected throughout the whole sample volume. In PHEMA polymerizing in 80 vol.% water, the size of a sphere is approximately between 4 and 8 μm. The thorough description of the phase separation phenomenon in forming gels and examples of resulting morphologies in methacrylate hydrogels can be found in [19,24,36,37] and an example of a PHEMA sphere-like hydrogel formed by reaction induced phase separation, H80/1, will be given in Part 3.3 below.

It was found earlier by Refojo [26] that in lightly crosslinked poly(glycerol monomethacrylate) systems (containing below 0.5 wt% of a bi-vinyl crosslinker) and diluted up to approx. 90 vol.% water in the monomer solution, the gels still absorbed more water after polymerization—a clear indication that PGMA gels did not phase separate during their formation. Indeed, the presence of a diluent during network formation alters the hydrogel macromolecular network topology—with more dilution, more elastically inactive loops are formed on the polymer backbone and less chains contribute to network elasticity, thus the final network is more loose, more swellable and it exerts lower modulus of elasticity than a network formed in the absence of a diluent [38]. The dilution of the GMA-based system in our experiment was 40 vol.% so the water level was far below the phase separation critical dilution and the gel was macroscopically homogeneous.

The equilibrium swelling of G40/0.3 hydrogel at room temperature corresponded approx. to 85 wt.% water (EWC 5.8 g/g) while for G0/0.3 the water content was 74 wt% (EWC 2.8 g/g), cf. swelling values of all tested gels in Table 1. 

**Figure 4:**
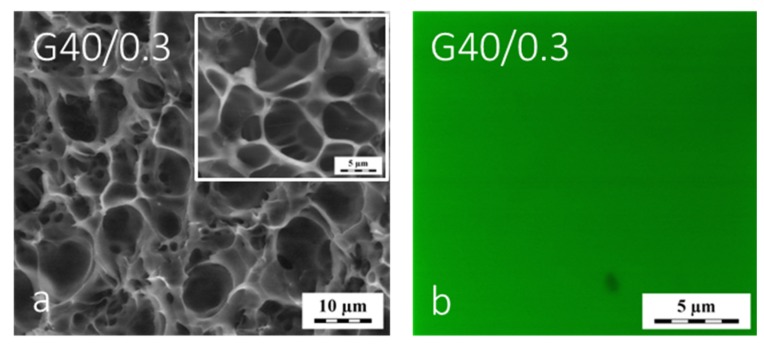
Cryo-SEM micrograph (**a**) and LSCM micrograph (**b**) of the water-swollen G40/0.3 hydrogel.

This difference in swelling well marks the difference of the macromolecular networks in G40/0.3 vs. G0/0.3 caused merely by network formation in the presence of various amounts of diluent. The G40/0.3 hydrogel was optically transparent in all states: as-prepared, equilibrium-swollen—expanded and dried at elevated temperature. The cryo-SEM images of the water-swollen G40/0.3 gel revealed porous cellular-like structure shown in Figure 4a. This structure is very similar to that of gel G0/0.3, cf. Figure 1e,f. In the sample G40/0.3, the porous structure revealed by cryo-SEM was attributed to the formation and sublimation of ice crystals following the plunge-freezing (in LN_2_). However, the observed morphology of G40/0.3 was somewhat different, revealing the number of small round-shape voids in the walls of cellular cavities. When the swollen gel G40/0.3 was chemically labeled by FITC and investigated by LSCM, there was no structure on the level of tens of micrometers seen, cf. Figure 4a vs. Figure 4b. The major difference between the G0/0.3 and G40/0.3 gels was thus their swollen macromolecular network topology resulting in a considerably different swelling degree and equilibrium modulus of elasticity, 250 kPa vs. 30 kPa (Table 1) respectively. The change of morphology of these hydrated gels when exposed to freezing conditions was qualitatively still similar, compare Figure 1e,f and Figure 4a,b.

### 3.3. PHEMA Hydrogels

Covalently crosslinked macromolecular hydrogels based on poly(2-hydroxyethyl methacrylate), PHEMA, swell in water significantly less compared with the gels based on PGMA, cf. Table 1. For example, PHEMA hydrogel prepared without diluent absorbed 0.6 g of water per g of dry matter in equilibrium while EWC of G0/0.3 was 2.8. Although PHEMA is chemically rather similar to PGMA, its chains are less hydrophilic: they attain a special state of equilibrium conformation in water achieving balance between hydrophilic and hydrophobic interactions as described in [24]. We prepared PHEMA hydrogels in the absence as well as in the presence of a water diluent: sample H0/0.3 and sample H40/0.3. Both hydrogels as prepared and swollen to equilibrium volume in water were expectedly transparent. In their dry state, they revealed homogeneous morphology by ESEM, LM and cryo-SEM—similarly as for PGMA hydrogels (Figure 1a,b; the images for dry PHEMA not shown).

**Figure 5:**
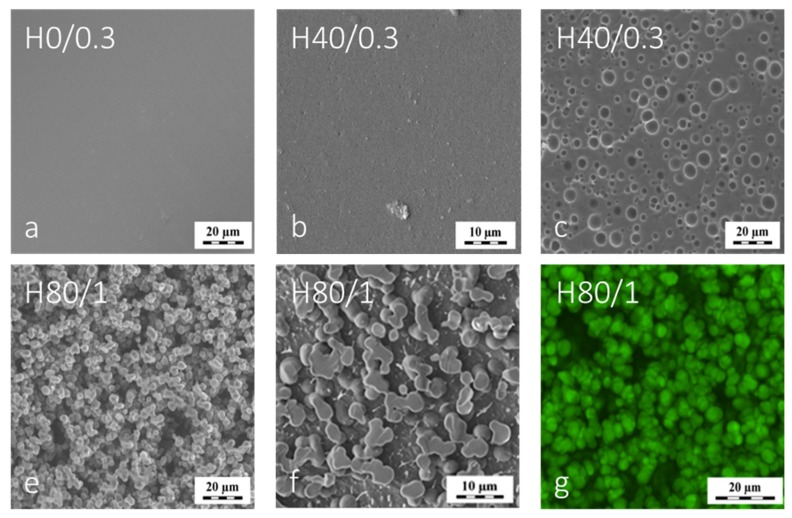
Micrographs of various hydrogels prepared from lightly crosslinked PHEMA. Figures a,b,c show microstructure of macroscopically clear gels by cryo-SEM and HVSEM: (**a**) cryo-SEM of H0/0.3 and (**b**,**c**) HVSEM and cryo-SEM of H40/0.3 produced below critical water dilution. Figures (**d**,**e**,**f**) show micrographs of H80/1 hydrogel by (**d**) cryo-SEM, (**e**) HVSEM and (**f**) LSCM.

Contrary to G0/0.3, the swollen bulk-prepared H0/0.3 hydrogel revealed a homogeneous, non-macroporous structure when treated by plunge-freezing in the same way as G0/0.3 for the cryo-SEM investigation, cf. Figure 5a. We explained this different morphology by the much lower content of water in PHEMA gel (EWC_H0/0.3_ = 0.6 g/g compared with EWC_G0/0.3_ = 2.8 g/g). Clearly, the water phase in the PHEMA gel was interacting with the macromolecular network to a much higher extent and thus water did not freeze out under the plunge-freeze conditions. Interestingly, the H40/0.3 swollen gel also attained a low amount of water (EWC_H40/0.3_ = 0.7). This gel revealed a uniform non-porous structure (in given resolution range) by HVSEM, see Figure 5b but incipient porous morphology was revealed by the cryo-SEM of H40/0.3, see Figure 5c. However, the number of pores over the H40/0.3 frozen surface observed at the same exposition time was significantly lower than that in PGMA hydrogels, compare Figure 4a and Figure 5c.

The PHEMA macroporous hydrogels were produced by phase separation occurring above the critical water content that is approx. 45–50 vol.% for lightly crosslinked PHEMA. Thus, the gel H80/1 made at 80 vol.% of water falls in the range where the reaction-induced phase separation occurs for thermodynamic reasons as well described in the past [19,20]. The forming PHEMA gel matrix formed the typical fused-sphere-morphology while the voids between the spheres formed a continuous porous space as revealed by SEM and LM and shown in Figure 5d,e,f. The equilibrium water content in H80/1 was 4.4 g/g, which was relatively high compared with the swelling of H0 and H40 gels (cf. Table 1). The EWC_H80/1_ corresponded to the dilution by water during reaction, which was 4.3 g of the water diluent per g of monomers. This means, that the overall volume of the formed structure is almost unchanged while the gel matrix in forming H80/1 forms the gel spheres expelling some water into the interstitial space. In the swelling equilibrium of H80/1, certain portion of water is located in the space between the gel spheres while some water swells the spheres. Thus, the swelling of the hydrogel phase in H80/1 must be significantly less than EWC = 4.4 g/g. The swelling of spheres was previously found to be close to swelling of H40/0.3, EWC_Hspheres_ = 0.7 [24]. This suggests that the gel in the spheres was not changed under the conditions of plunge-freezing and cryo-SEM and behaved similarly as the gel H40/1. Moreover, comparison of the morphological structure of H80/1 by cryo-SEM with that by LSCM and even by HVSEM showed that the morphology of H80/1 seen by cryo-SEM was not significantly distorted, see Figure 5d,e,f. Clearly, the spatial arrangement of the gel spheres and their flexible connections could facilitate accommodation of the possible solid ice formed upon plunge-freezing in the LN_2_.

### 3.4. IPN Hydrogels

**Scheme 2:**
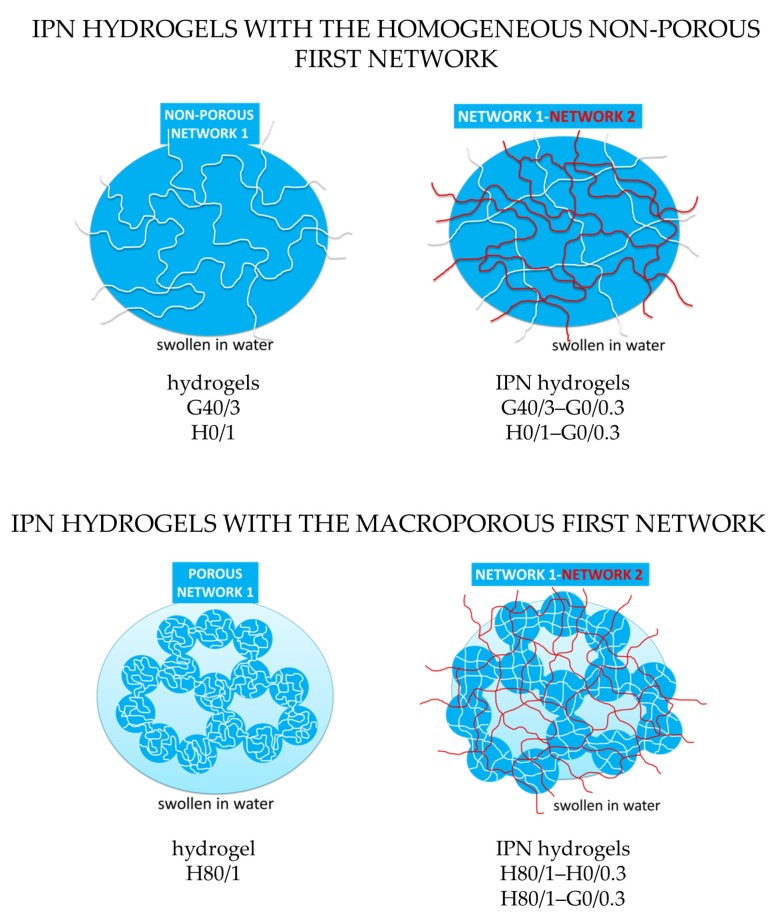
Distribution of the first and second macromolecular networks in interpenetrating network (IPN) hydrogels prepared by sequential polymerization presented schematically. The produced homogeneously interpenetrated networks based on G40/3 and H0/1 and microstructured IPNs based on H80/1.

We have also investigated much stiffer swollen hydrogels made by reinforcing the primary hydrogel network (network 1) with the second, chemically similar interpenetrating network 2. The IPN hydrogels were made by sequential polymerization of network 2 components swollen in network 1. As the first networks, either homogeneous, non-porous (G40/3, H0/1) or heterogeneous, phase-separated (H80/1) matrices were chosen. The preparation of such gels and their morphologies are schematically shown in Scheme 2, details of the preparation were elaborated by Sadakbayeva et al. [39]. The morphological microstructure of the water-swollen IPN gels was investigated by cryo-SEM. Again, the images reflected the capacity of water to form crystals in the non-porous gels at plunge-freezing in LN_2_ and following low temperature treatment in the microscope. Let us consider the example of two IPN hydrogels with macroporous network significantly differing in water content: 1/IPN hydrogel H80/1-H0/0.3 that contains 38 vol.% of water and 2/IPN hydrogel H80/1-G0/0.3 that contains 70 vol.% of water (cf. Table 1).

**Figure 6:**
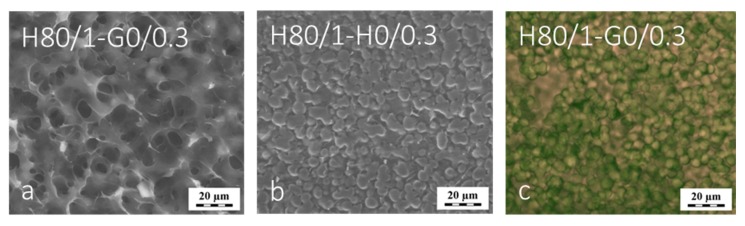
Cryo-SEM (**a**,**b**) and LSCM micrographs (**c**) of IPN hydrogels made from the macroporous first network H80/1—IPNs composition printed within images.

The used macroporous matrix was the same in both cases, yet its state during the polymerization of network 2 was somewhat different due to different swelling in the GMA monomer (ESC_H80/1_ for HEMA was 10.2 g/g and for GMA 13.0 g/g). High swelling of network 1 in both monomers suggested that its polymer phase was evenly penetrated with the monomer of network 2 like depicted in the Scheme 2. The EWC of the resulting structured IPNs was different (2.2 g/g for H80/1-G0/0.3 and 0.6 g/g for H80/1-H0/0.3). Thus the possibility of “secondary” formation of pores during cryo-SEM was more likely for the system H80/1-G0/0.3. Indeed, in the H80/1-G0/0.3 macroporous morphology was found by cryo-SEM: the boundaries of spherical particles of the first gel were partially preserved and embedded in second network, but numerous holes in both phases were clearly visible (Figure 6a). The H80/1-H0/0.3 sample provided a clearly distinguished two-phase area with visible gel fused spheres and material filling the space around the spheres, both phases showing no porosity (Figure 6b). The mechanical failure at the cryo-SEM experiment of the gel spheres in the H0/1-G0/0.3 hydrogel was investigated in detail by the experiment (see Supplementary data, Figure S3).

Finally, the morphology of the microstructured IPN (MIPN) hydrogels in their native swollen state was elucidated using LSCM. This method was already applied for various IPN systems [33,40,41,42]. To distinguish the two networks in the IPN hydrogel, we introduced the two different modified dyes (the methacryloylated fluorescein—green color and DY-677—red color) in both monomer mixtures of H80/1-G0/0.3 at polymerization. Different light absorption and emission properties of these dyes allowed distinct observation of the corresponding hydrogel phases, see Figure 6c. The image shows that the H80/1-G0/0.3 hydrogel consists of fused spherical regions of mixed color: the gel spheres should contain both PHEMA (labeled by methacryloylated fluorescein, green color) and PGMA (labeled by DY 977, red color) separated by the regions containing only PGMA (network 2 labeled by red color); no polymer-free voids were identified with LSCM in this IPN hydrogel. Thus, the porous morphology evident in Figure 6 developed during cryo-SEM as a secondary structure and is considered artifactual.

### 3.5. Effect of Plunge-Freezing at Cryo-SEM on the Hydrogel Microstructure

As it was shown with the H40/0.3 homogenous sample, the swelling capacity alone cannot serve as a single predictive parameter for assessing the possibility of “secondary porosity” formation induced by the freezing cycle. For example, the H40/0.3 sample revealed the artificial porous structure despite its low equilibrium swelling in water (EWC_H40/0.3_ = 0.7 g/g). We constructed an empirical map of the cryo-SEM behavior of hydrogels studied here in order to understand the limits of this popular technique and to set semi-predictive criteria of the applicability of plunge-freezing with cryo-SEM for correct visualization of the swollen gels morphology without artifacts.

**Figure 7:**
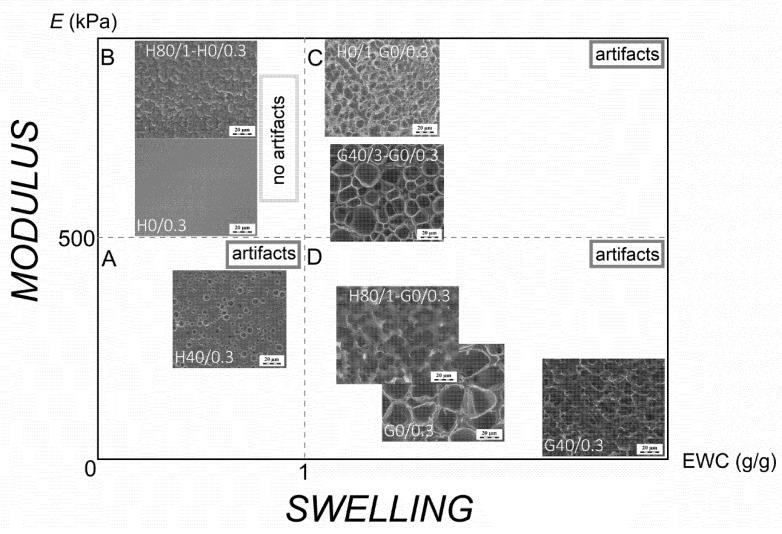
A cryo-SEM “map” of studied non-porous hydrogels: Young’s modulus (*E*) vs. equilibrium water content (EWC).

In the schematic map on Figure 7, the hydrogels were arranged according to their tensile modulus (*E*) as a mechanical parameter along the ordinate and their equilibrium water content along the abscissa. Four segments (A–D) in the map are related to the gels with relatively high or low values of *E* and EWC, which indicate the reliability borders of the plunge-freezing cryo-SEM method. According to these schematics, the cryo-SEM observation gave relevant results exclusively for the samples from segment B: those with low EWC and high *E*. When the EWC was too high (segments C and D), the formed ice crystals led to the appearance of secondary porosity irrespectively of the gel mechanical strength. When the tensile modulus of the gel was too low (segment A), the matrix could not withstand the formation of ice crystals even at relatively low swelling capacity.

The pore size of PGMA-based hydrogels revealed by means of cryo-SEM observation was little dependent on the position of the sample in the map. Moreover, the pore size distribution was quite wide (Table 1). Finally, the observed pore size may be a function of observation time (Figure 3). The pores appearing in the H40/0.3 gel were significantly smaller as compared to the PGMA gels, due to the lower equilibrium water content of the PHEMA gel.

In conclusion, it should be noted that the empirical two-variable criterion could be applied to the multiphase samples (such as MIPNs). As shown for the macroporous H80/1 sample (for the structure see Figure 5f), no secondary pores were found by the cryo-SEM images in the gel spheres, even though its overall EWC was relatively high (4.4 g/g) and its apparent Young’s modulus was very low (4 kPa). In the case of such heterogeneous systems, rather the hydrogel phase properties should be considered but these may not be easily experimentally accessible.

## 4. Conclusions

Morphological structures of a series of reference methacrylate-based hydrogels with varied water content and starting morphology was investigated. It was shown that porosity in swollen poly(glycerol monomethacrylate) (PGMA) hydrogels revealed by cryo-SEM was artifactual as confirmed by direct observation of the same swollen hydrogels at depressed temperature by ESEM or in ambient conditions by light microscopy (LM) and laser scanning confocal microscopy (LSCM) of labeled samples. Clearly, the multiple and homogenously dispersed pores evident in PGMA gels in cryo-SEM images were formed during the sample plunge-freezing step and subsequent low temperature conditions during the image acquisition.

Within the hydroxymethacrylate-based hydrogel series, we found a relation between the hydrogels water content and their tendency to show artifacts in cryo-SEM and HVSEM. We proposed to consider two hydrogel parameters: swelling degree and mechanical stiffness expressed using elastic moduli as the criteria for the assessment of applicability for cryo-SEM studies of water-swollen hydrogels. These criteria were investigated with a series of hydrogels of the analogous chemical composition but with varied morphology: homogenous gels, macroporous gels, homogeneous interpenetrating network gels and microstructured interpenetrating network gels and found matching with the occurrence of artifacts by cryo-SEM (or HVSEM). It follows that morphological structure of highly swollen and mechanically weak hydrogels cannot be assessed solely by using cryo-SEM or HVSEM, which involve sample freezing. These structures should always be confirmed by microscopy techniques applicable for gels in their swollen state. In this work, we confirmed the applicability of wide-field LM, LSCM and ESEM.

## Figures and Tables

**Table 1:** Parameters of investigated gels. Visual appearance in equilibrium water-swollen state of gels, equilibrium water content (EWC), Young’s modulus (*E*) and the size of pores detected by cryo-SEM.

Sample	Visual Appearance of Water-Swollen Gel	EWC (g/g)	Size of Pores in Cryo-SEM Image (μm)	*E* in Swollen State (kPa)
H0/0.3	clear	0.6	no pores	590
H40/0.3	clear	0.7	0.6–10	430
H80/1	white	4.4	2–5	4
G0/0.3	clear *	2.8	2–40	250
G40/0.3	clear	5.8	2–25	30
H80/1-H0/0.3	opaque	0.6	no pores	980
H80/1-G0/0.3	opaque	2.2	7–30	380
G40/3-G0/0.3	clear *	1.9	2–25	540
H0/1-G0/0.3	clear *	1.1	2–30	1010

* very slight scatter from a water-swollen sample, cf. Figure 1c.

## Supplementary Materials

The following are available online at https://www.mdpi.com/2073-4360/12/3/578/s1, Figure S1. Micrographs from cryo-SEM made with water-swollen G0/0.3 hydrogels prepared by UVphotopolymerization using: (a, b) DEGDMA crosslinker and (c) GDMA crosslinker. The monomers were used without purification (a) or were preliminary purified by distillation and degassed (b, c). UV irradiation in the case (a) was accompanied by additional heating (T = 120 °C), Figure S2. ESEM micrographs of water-swollen PHEMA hydrogel (H60/5) prepared at 60 wt.-% of water and 5 mol-% of DEGDMA crosslinker. The morphology of sample was observed under various pressures ( 630 - 340 Pa), Figure S3. Cryo-SEM micrographs of water-swollen (a) H0/1-H0/0.3 and (b) H0/1-G0/0.3 interpenetrating network hydrogels.
