# Supplementary material for: Microscopic Structure of Swollen Hydrogels by Scanning Electron and Light Microscopies: Artifacts and Reality
Source: Polymers (Basel). 2020 Mar 5;12(3):578. doi: 10.3390/polym12030578 (PMC7182949; doi:10.3390/polym12030578)
Supplement: Supplementary file 1 [file polymers-12-00578-s001.pdf]

Supporting Information

Microscopic structure of swollen hydrogels by scanning electron and light microscopies. Artifacts and reality

Zhansaya Kaberova[a], Evgeny Karpushkin[b], Martina Nevoralová[a], Miroslav Vetrík[a], Miroslav Šlouf[a], and Miroslava Dušková-Smrčková[a]*

[a]*Institute of Macromolecular Chemistry, Academy of Sciences of the Czech Republic, Heyrovského náměstí 2, 162 06 Praha 6, Prague, Czech Republic*

[b]*Department of Chemistry, Lomonosov Moscow State University, Moscow, Russia*

[*]Corresponding author.

E-mail: m.duskova@imc.cas.cz

Hydrogel synthesis

a) Synthesis of bulk hydrogels

Reference PGMA and PHEMA hydrogel were synthesized by photopolymerization of a reaction mixture containing GMA or HEMA monomer, 0.5 mol-% (with respect to the monofunctional monomer) of DEGMA crosslinker, and 0.3 wt.-% (with respect to both monomers) of 2-methylpropiophenone (DAROCUR 1173) photoinitiator. The reaction mixture was stirred for 5 min at RT, bubbled with dry nitrogen, injected between two glass slides separated by a silicone rubber spacer and exposed to UV radiation with the intensity of 10 mW/cm^2 for 90 min. After the polymerization, the samples were always thoroughly washed with distilled water to remove unreacted monomers and possible other soluble residues. The reaction mixture was heated up to T = 120°C during the UV irradiation. Free-radical photopolymerization of GMA is an exothermal reaction, and in the absence of inert diluent the evolved specific heat is fairly high: when the reaction was initiated at room temperature, the mixture was self-heated to about 75°C within 10 minutes. Such heating might cause boiling of the reaction mixture and lead to the formation of microbubbles. On the

other hand, after 10 minutes of polymerization, the temperature abruptly dropped down to 40–45°C, which was significantly below the glass transition temperature of the formed polymer[24,25], the chains can loose their segmental mobility, and the vitrification sets in. In glassy state, some microbubbles can be trapped inside the prepared sample and this could explain the morphology found in the cry-SEM micrographs-(Fig. 3a). Thus, the additional heating to $T = 120$°C during the reaction was used to keep the forming polymer above its glass transition temperature (that was about 109°C determined by DSC) and thus facilitating the bubbles removal. However, the cryoSEM image obtained for the thus prepared sample for the swollen gel prepared by photopolymerization at $T > T_g$ revealed porous structure as well.

b) Synthesis including freeze-pump-thaw-purge cycles.

The reaction mixture containing GMA monomer, crosslinker (DEDGMA or GDMA) at a level of 0.5 mol-% (in monomers) and 0.3 wt.-% (in monomers) of 2-methylpropiophenone (DAROCUR 1173) photoinitiator went through the three freeze-pump-thaw-purge cycles on the Schlenk line to remove dissolved gases. Each cycle included 5 min of freezing in dry ice/ethanol bath, 15 min of pumping, 15 min of thawing in a tepid water bath, and 15 min of purging with argon. Once the reaction mixture was purified and degassed, it was quickly transferred into the mold under the inert atmosphere and exposed to UV of 10 mW/cm^2 intensity during 90 min. After the photopolymerization, the gel was extensively washed with distilled water.

The reaction mixture was degassed using the Schlenk line prior to polymerization. The idea was as follows: mere purging of the reaction mixture with nitrogen to remove the dissolved oxygen could be accompanied by redissolution of nitrogen enhancing the bubbles formation. However, degassing on the Schlenk line, due to additional freezing and thawing of the mixture under vacuum, pumps away the non-condensable gases. Efficiency of the freeze-pump-thaw-purge method is improved when each cycle is repeated several times. However, the PGMA samples prepared in this study via the photopolymerization after three purification cycles, still exhibited pores in the cryoSEM images (Fig. 3b).

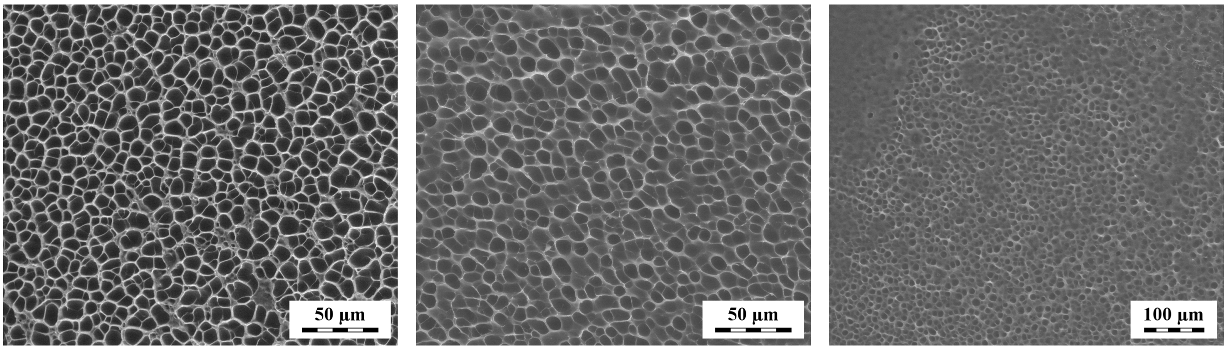

Figure S1. Micrographs from cryo-SEM made with water-swollen G0/0.3 hydrogels prepared by UVphotopolymerization using: (a, b) DEGDMA crosslinker and (c) GDMA crosslinker. The monomers were used without purification (a) or were preliminary purified by distillation and degassed (b, c). UV irradiation in the case (a) was accompanied by additional heating (T = 120 °C).

c) Synthesis of hydrogels in the presence of water as diluent

The reaction mixture containing a monomer (HEMA or GMA), 40 vol.-% or 80 vol.-% of water, 1 mol-% (with respect to HEMA or GMA) of DEDGMA crosslinker, 0.75 wt.-% (with respect to both monomer) of APS initiator, and TEMED catalyst (5 μL of 5 wt.-% aqueous solution per 5 g of the mixture) was stirred for 5 min, bubbled with nitrogen, injected between two glass slides separated by a silicone rubber spacer and left overnight at room temperature for complete polymerization. The prepared gels were thoroughly washed with distilled water.

Finally, we changed the crosslinker type: GDMA was used instead of DEGDMA. The idea behind was as follows: GDMA contained an additional hydroxyl group instead of ether group, and hence was more hydrophilic and thus possibly more miscible with GMA monomer. Thus, we expected GDMA units uniformly distributed over the network and not causing assemby of nanodomains during the polymerization. On top of that, similarly to case 2 above, the reaction mixture containing purified GMA monomer, GDMA crosslinker, and DAROCUR 1173 initiator was degassed using freeze-pump-thaw-purge method. However, the improvement in the comonomers compatibility did not lead to the formation of nonporous gel as observed by cryoSEM (Fig. 3c).

Synthesis of IPN hydrogels

Macroporous PHEMA network (Network I) was synthesized by redox-initiated radical polymerization in an aqueous solution containing 80 vol.-% of water, 1 mol-% (with respect to HEMA) of DEGDMA crosslinker, 0.75 wt.-% (with respect to both monomers) of APS initiator and TEMED catalyst (5 μL of 5 wt.-% aqueous solution per 5 g of the mixture). Polymerization reaction was run overnight at room temperature. The unreacted monomers were removed from the hydrogel by washing. The water-swollen PHEMA gel was immersed into the reaction mixture for the second network (Network II) preparation, containing the monomer (HEMA or GMA), 0.3 mol-% (with respect to HEMA or GMA) of DEGDMA crosslinker and 0.5 wt.-% (with respect to both monomers) of DAROCUR 1173 initiator. Water in the hydrogel was gradually replaced with the monomers by exchanging the reaction mixture with fresh portion several times after equilibration. The absence of water in the system was confirmed by ATR FTIR method and measuring the refractive index of the liquid. Once the equilibrium swelling was achieved, the solid PHEMA network swollen in the reaction mixture was irradiated by UV with intensity of 10 mW/cm^2 during 90 min and then thoroughly washed with distilled water to remove the unreacted monomers.

Preparation of fluorescently-labelled hydrogels

A single-network PGMA hydrogel was labelled by incorporating the vinyl- modified fluorescein added directly to the monomer reaction mixture (approx. 0.001 wt.-%). The synthetic procedure of fluorescein modification is described below in Supplementary data. In the case of IPNs, each network was labelled using the modified dyes with different excitation wavelengths in order to distinguish between the two networks. The first (PHEMA) network was labelled with the modified fluorescein, while the second (PGMA) one was labelled with the modified dye DY-677. Concentration of the dyes in both cases was 0.001 wt.-%. After the polymerization of each network, the excess of unreacted dye and monomers was removed by extensive washing of the gel with distilled water until the washings became visually colorless.

Modification of fluorescent dyes

Methacryloylation of DY-677-NHS

0.5 mg (489.2×10^{-9} mol) of DY-677-NHS, an amine-coupling asymmetric cyanine ester with excitation spectrum peak at 673 nm, in 500 µL of dimethylformamide was added dropwise to a stirred solution of *N*-(3-aminopropyl)methacrylamide hydrochloride (0.131 mg, 0.73×10^{-9} mol) with 5 µL of triethylamine. The reaction was carried out for 24 h at room temperature. The reaction mixture was evaporated under vacuum and purified on a silica gel column with acetone–H_2O = 9:1 as the mobile phase. After the purification, the blue-colored product was lyophilized for storage.

Methacryloylation of fluorescein

Fluorescein isothiocyanate ester (0.5 g, 1.3 mmol) with excitation spectrum peak at 495 nm was dissolved in 100 mL of anhydrous tetrahydrofuran, and 0.215 mL (1.5 mmol) of triethylamine was added to the solution. The mixture was cooled to 0°C, and 0.146 mL (1.5 mmol) of methacryloyl chloride in 5 mL of tetrahydrofuran was added dropwise. The mixture temperature was kept around 0°C during the mixing, and then the addition reaction was carried out at r.t. for 24 h. Tetrahydrofuran was evaporated off, and the product was purified by column chromatography with $CHCl_3$–acetone = 9:1 as the mobile phase.

Effect of variable pressure during the environmental scanning electron microscopy

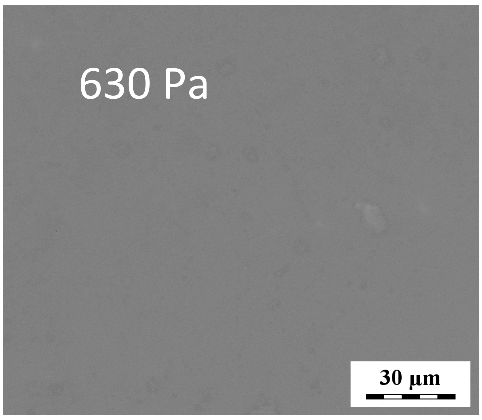

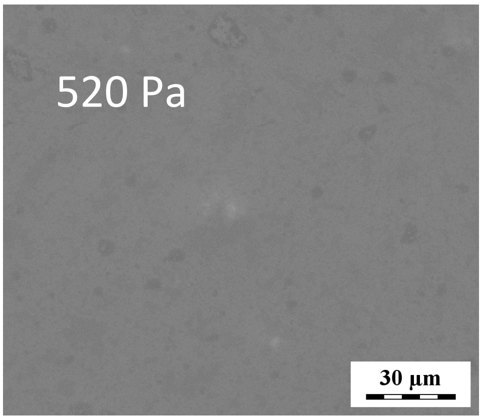

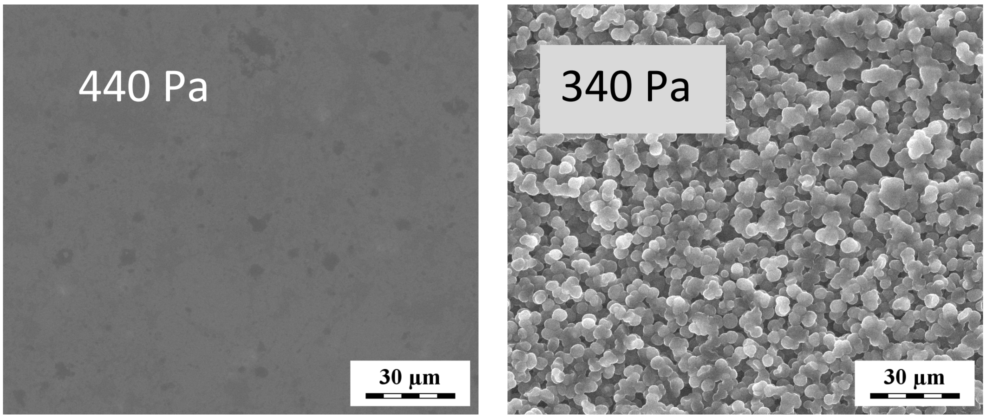

Figure S2. ESEM micrographs of water-swollen PHEMA hydrogel (H60/5) prepared at 60 wt.-% of water and 5 mol-% of DEGDMA crosslinker. The morphology of sample was observed under various pressures (630 - 340 Pa).

Morphology of swollen IPN hydrogels by cry-SEM

The homogeneously interpenetrated single-phase H0/1-G0/0.3 and H0/1-H0/0.3 IPNs were prepared using a nonporous H0/1 matrix as first network. The second network was either the H0/0.3 or G0/0.3.The cryo-SEM micrographs for those IPNs are shown in Fig. S3. The H0/1-G0/0.3 sample shows highly porous morphology, cf. Fig. S 3a. The H0/1-H0/0.3 hydrogel did not reveal pores by the cryo-SEM treatment (Fig. S3, b) - its swelling capacity in water was rather low (EWC 0.57 g/g).

It is interesting to notice that the size of the pores formed H0/1-G0/0.3 sample was the same as for the single G0/0.3 hydrogel (e.g., Fig. 1). Hence, the presence of first PHEMA network virtually did not restrict formation of the porous structure, yet affecting other properties like overall swelling (cf. $EWC_{G0/0.3}$ = 2.8 g/g vs. $EWC_{H0/1\text{-}G0/0.3}$ = 1.1 g/g).

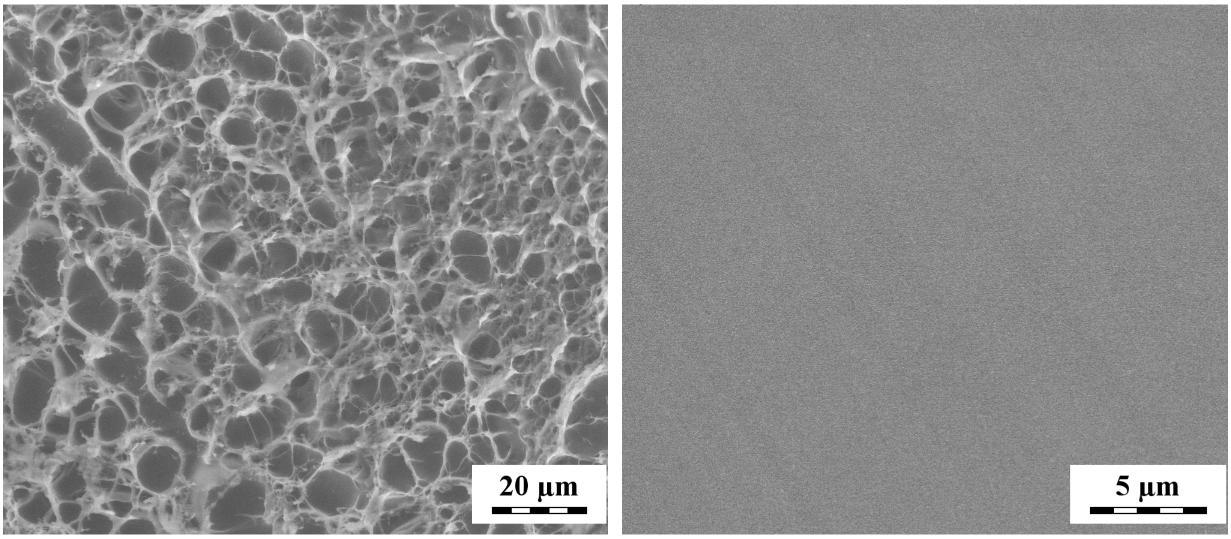

Figure S3. Cryo-SEM micrographs of water-swollen (a) H0/1-H0/0.3 and (b) H0/1-G0/0.3 interpenetrating network hydrogels.
